# Explainable CNN–BiLSTM Framework for Multi-Class Sleep Apnea Severity Detection Using Single-Lead ECG Signals: A Comprehensive Machine Learning Approach

**DOI:** 10.3390/diagnostics16152353

**Published:** 2026-07-27

**Authors:** Fida’a Al-Quran, Malik Jawarneh, Omar Isam AL-Mrayat, Dyala Ibrahim, Ghassan Samara, Alaa Sheta, Ghada Elmarhomy, Nadiah A. Baghdadi, Amer Malki, El-Sayed Atlam

**Affiliations:** 1 Engineering and Artificial Intelligence Department, Al-Salt Technical College, Al-Balqa Applied University, Al-Salt 19117, Jordan; fmalquran@bau.edu.jo; 2 Department of Computer Science, College of Information Technology, Amman Arab University, Amman 11953, Jordan; 3 Department of Software Engineering, Amman Arab University, Amman 11953, Jordan; o.mrayat@aau.edu.jo; 4 Departments of Computer Science, Middle East University, Amman 11831, Jordan; d.ibrahim@meu.edu.jo; 5 Department of Computer Science, Faculty of Information Technology, Zarqa University, Zarqa 13132, Jordan; gsamara@zu.edu.jo; 6 Computer Science Department, School of Information Technology and Computer Science, Nile University, El Sheikh Zayed City 12677, Egypt; 7 Computer Science Department, Southern Connecticut State University, 501 Crescent St., New Haven, CT 06515, USA; 8 Department of Information Systems, College of Computer Science and Engineering, Taibah University, Yanbu 46421, Saudi Arabia; gmarhomy@taibahu.edu.sa; 9 Department of Nursing Management and Education, College of Nursing, Princess Nourah bint Abdulrahman University, Riyadh 11671, Saudi Arabia; nabaghdadi@pnu.edu.sa; 10 Department of Computer Science, College of Computer Science and Engineering, Taibah University, Yanbu 46421, Saudi Arabia; asamalki@taibahu.edu.sa (A.M.); or satlam@yahoo.com (E.-S.A.); 11 Department of Computer Science, Faculty of Science, Tanta University, Tanta 31527, Egypt

**Keywords:** obstructive sleep apnea, deep learning, explainable artificial intelligence, electrocardiogram, heart rate variability, SHAP, LSTM, CNN, sleep disorders, automated diagnosis

## Abstract

**Background/Objectives:** Obstructivesleep apnea (OSA) is one of the most widespread forms of sleep disease, affecting over 936 million adults globally. The health consequences of obstructive sleep apnea (OSA) are well documented; however, it remains largely underdiagnosed because the current gold-standard diagnostic method, polysomnography (PSG), is often costly, time-consuming, and unavailable in many healthcare settings. To address these challenges, this study presents a novel explainable deep learning (DL) framework for automated multi-class OSA severity classification using single-lead electrocardiogram (ECG) signals. **Methods:** The proposed framework integrates a hybrid CNN–BiLSTM architecture with explainable artificial intelligence (XAI) techniques to generate clinically meaningful predictions and explanations across four OSA severity classes: Normal, Mild, Moderate, and Severe. The framework was evaluated using the publicly available PhysioNet Apnea-ECG dataset (70 recordings) together with an institutional ECG dataset (150 recordings), resulting in a combined cohort of 220 recordings. **Results:** The proposed framework achieved an overall classification accuracy of 94.7%, with sensitivity and specificity values of 92.3% and 96.1%, respectively. Furthermore, the proposed model consistently outperformed conventional machine learning algorithms, including Support Vector Machine (SVM), Random Forest, and XGBoost, by 5.5%, 4.2%, and 2.9%, respectively. To enhance transparency and clinical trust, SHAP (SHapley Additive exPlanations) was employed to identify the most influential physiological predictors driving model decisions. Heart rate variability features, particularly RMSSD and pNN50, emerged as the strongest indicators of OSA severity. Moreover, computational efficiency analysis revealed that the model required only 0.23 s to process a 60 s ECG epoch on a standard computing platform, supporting its suitability for real-time deployment. **Conclusions:** The findings demonstrate that explainable deep learning applied to ECG signals can provide accurate, interpretable, and computationally efficient assessment of OSA severity. The proposed framework may support OSA screening, clinical triage, and early intervention, particularly in resource-constrained healthcare environments.

## 1. Introduction

### 1.1. Background and Clinical Significance

Obstructive sleep apnea (OSA) is a long-term condition of a sleeping disorder related to breathing that is marked by periodic blockage attributed to the upper airway during sleep, resulting in intermittent hypoxia, sleep fragmentation, and a considerable cardiovascular burden [1,2,3].

According to epidemiological research, it is estimated that there are almost 936 million adults across the world who experience some obstructive sleep apnea, with moderate and severe cases being observed in approximately 425 million people all over the world [4]. OSA is still growing because of the increasing number of obese people, aging, and risk factors related to lifestyle [5].

The medical implications of OSA in untreated individuals go way beyond bad sleep. There is a close relationship between OSA and hypertension, coronary artery disease, stroke, type 2 diabetes, and all-cause mortality [4]. The discontinuous hypoxia and sympathetic stimulation of OSA events also lead to endothelial dysfunction, systemic inflammation, and metabolic dysregulation [5]. Moreover, sleep fragmentation leads to excessive daytime drowsiness that considerably deteriorates cognitive functioning and productivity at work and predisposes to the occurrence of motor vehicle accidents [6].

Though OSA is a serious disease with significant health outcomes, it is terribly under-diagnosed. It is approximated that no less than 80–90% of adults with clinically significant OSA are not diagnosed [7]. This diagnostic gap can be mainly explained by the weaknesses of polysomnography (PSG), which is the gold standard of OSA diagnosis. PSG requires overnight monitoring in specialized sleep laboratories with multiple physiological sensors, is expensive, time-consuming, and uncomfortable for patients, and has limited availability, particularly in rural and underserved areas [8,9].

### 1.2. Alternative Diagnostic Approaches and ECG-Based Detection

In order to overcome the drawbacks of PSG, scientists have considered other types of diagnostic modalities, such as home sleep apnea testing (HSAT), wearable devices, and single-channel physiological signals [10]. Of these methods, ECG-based OSA recognition has been most actively pursued because of the following strengths: (1) ECG recording is non-invasive, readily accessible and can be recorded using portable or wearable devices; (2) single-lead ECG recordings offer more benefits with respect to patient discomfort and equipment expenses; (3) ECG signals have rich physiological content reflecting the autonomic arousal of the nervous system to apneic events; and (4) ECG infrastructure is currently widely prevalent in primary care environments, which can easily be translated into extensive use.

ECG-based OSA detection is physiologically based on the cardiovascular response to apneic events. In apnea periods, the development of hypoxemia leads to compensatory mechanisms such as augmented sympathetic stimulation, constriction of peripheral vessels, and varying heart rate. These physiological alterations are reflected in ECG signals of heart rate variability (HRV), the morphology of the QRS, and ST-segment changes. These features derived through ECG can be used to differentiate between normal respiration and apneic events, and determine the severity of OSA [11,12,13].

### 1.3. Machine Learning and Deep Learning in OSA Detection

The use of machine learning (ML) and deep learning (DL) tools in automated OSA detection has changed considerably in the last 10 years. Conventional ML models, such as support vector machine (SVM), random forests, and gradient boosting have shown promising performances when used on handcrafted features derived from ECG signals. Such techniques usually produce accuracies of 85–92% (normal vs. apnea) in binary OSA classification.

The current developments in deep learning have made possible end-to-end learning models that can be used to automatically learn discriminative features based on raw or low-level processed ECG signals. Convolutional neural networks (CNNs) are researchers that do particularly well at identifying spatial information within ECG morphology, whereas recurrent neural networks (RNNs) and especially long short-term memory (LSTM) networks are at least as effective at identifying temporal patterns in sequential physiological data. Hybrid architecture defined as a combination of CNNs and LSTMs has been shown to perform better by taking advantage of the learning abilities (spatial and temporal features) of both the CNNs and the LSTMs.

Even with these technological advances, there are various challenges: (1) most studies concentrate on binary classification as opposed to clinically relevant multi-class severity grading; (2) black-box models used by deep learning make them less clinically acceptable; (3) generalizability across patient populations and ECG systems has not received much attention; and (4) real-time processing requirements in wearable deployment processes have had little attention.

Recent research has examined sophisticated deep learning designs to identify sleep apnea through physiological signals. Convolutional neural networks have demonstrated strong performance in extracting morphological features from ECG signals, while recurrent neural networks such as long short-term memory (LSTM) models effectively capture temporal dependencies in sequential physiological data. It has been demonstrated that hybrid CNN-LSTM networks are superior to traditional machine learning algorithms on a variety of biomedical signal classification classes. Moreover, explainable artificial intelligence methodologies have also been implemented recently in order to enhance the transparency and interpretability of deep learning models applied to healthcare systems [14,15,16,17,18].

Recent investigations regarding obstructive sleep apnea detection from ECG have revealed many advanced deep learning architectures for this purpose, which include transformer-based architectures, EfficientNet models, attention-based neural networks, etc., and explainable artificial intelligence approaches [19,20,21,22,23,24,25,26,27,28]. A large number of further recent works explored wearable ECG monitoring systems and SHAP-based interpretability techniques for clinical transparency and real-time applicability. In spite of this progress, many existing works keep focusing on binary apnea detection while offering less emphasis on clinically meaningful multi-class severity stratification, cross-dataset validation and deployment-oriented computational analysis. Motivated by these limitations, we propose an explainable CNN–BiLSTM framework for interpretable four-class OSA severity assessment using single-lead ECG signals.

### 1.4. Explainable AI in Medical Decision Support

Explainable artificial intelligence (XAI) methods are becoming perceived as essential to the clinical adoption of ML models. Healthcare providers need the ability to understand AI systems clearly and without interpretation to understand the reasons behind prediction being in agreement with clinical knowledge. SHAP (SHapley Additive exPlanations) is a popular technique that determines the contribution of every feature to a model prediction. SHAP analysis can be used in OSA detection to show what features of the HRV and ECG contribute to classification of the severity, which connects the outputs of ML with clinical interpretation.

### 1.5. Research Gap and Motivation

The existing literature shows important gaps: (1) most studies address binary or three-class classification, whereas clinical severity assessment requires four classes based on (Normal (AHI < 5), Mild (5 ≤ AHI < 15), Moderate (15 ≤ AHI < 30), and Severe (AHI ≥ 30)); (2) limited use of explainability methods tailored for clinical interpretation; and (3) single-dataset validation that can limit confidence in generalizability.

The proposed framework goes beyond the conventional ECG-based OSA studies that generally focus on binary apnea detection or end-to-end black-box deep learning architectures. Instead, the authors considered multi-class OSA severity stratification and physiological feature engineering for SHAP-based interpretability and cross-dataset validation within a lightweight explainable deep learning architecture. The study also fuses CNN–BiLSTM spatial–temporal learning with HRV and ECG morphological biomarkers, resulting in an improvement in classification interpretability and computational feasibility for wearable healthcare in real time. Also, the external cross-dataset validation combined with the explainability-driven physiological interpretation addresses the issues related to model transparency, generalizability and clinical applicability reported in previous studies on ECG-based OSA detection.

### 1.6. Research Objectives and Contributions

The results of this study can be summarized as the following contributions:

- We develop an explainable CNN–BiLSTM framework for four-class OSA severity classification using single-lead ECG signals.
- We integrate a CNN, BiLSTM and self-attention mechanism to extract spatial and temporal physiological features.
- We examine how SHAP-based explainability can help interpret predictors of OSA severity.
- We assess generalizability across different datasets and check the computational feasibility for real-time and wearable screening in healthcare applications.

### 1.7. Paper Organization

Section 2 describes datasets, preprocessing, feature extraction, model architecture, and experimental setup. Section 3 also provides performance results, explanatory analysis, cross-validation and computational efficiency. Section 4 contains clinical implications, limitations and future work. Section 5 concludes the paper.

## 2. Materials and Methods

### 2.1. Datasets

#### PhysioNet Apnea-ECG Database

The PhysioNet Apnea-ECG database is a widely used benchmark dataset for automated sleep apnea detection research. The database was originally introduced as part of the PhysioNet research resource for complex physiological signals and provides overnight ECG recordings annotated with apnea events. It contains 70 single-lead ECG recordings, each approximately eight hours in duration, sampled at 100 Hz with minute-by-minute apnea annotations. These annotations allow researchers to develop machine learning and deep learning models for automated sleep apnea detection and severity classification. Such data has been applied widely in the biomedical signal processing literature and is a standard test-bed in the analysis of sleep apnea detection algorithms based on ECG [29,30,31]. In this study, recordings were categorized according to apnea–hypopnea index (AHI) thresholds as Normal (AHI < 5, *n* = 10), Mild (5 < AHI < 15, *n* = 20), Moderate (15 < AHI < 30, *n* = 20), and Severe (AHI ≥ 30, *n* = 20).

The average number of apnea and hypopnea events per hour of sleep recording was used to compute the AHI values from the minute-by-minute apnea annotations. The standard clinical thresholds of AHI severity were used to categorize recordings as Normal, Mild, Moderate and Severe OSA.

The institutional dataset comprises ECG recordings obtained from adult subjects, who underwent supervised sleep assessment procedures, under the approved ethics. Only customized clinical ECG monitoring systems were used for recording. Participants were excluded from analysis due to severe corruption of the signal, incomplete annotations, or major arrhythmia [16,17,32,33]. Table 1 reflects other demographic and clinical characteristics.

### 2.2. Data Preprocessing

#### 2.2.1. Signal Quality Assessment

The recordings were divided into 60 s epochs. Signal quality testing incorporated baseline wander analysis, disconnection/saturation analysis, SNR estimation and R-peak detectability tests with the Pan–Tompkins algorithm. Epochs with SNR < 10 dB or R-peak detection failure were excluded, yielding 98,040 valid epochs (PhysioNet: 33,600; Institutional: 64,440).

#### 2.2.2. Noise Reduction and Filtering

A multi-stage filtering pipeline removed powerline interference using notch filtering (Q = 30), eliminated baseline wander using median filtering (200 ms) followed by a 4th-order high-pass Butterworth filter (0.5 Hz), and attenuated high-frequency noise using a 4th-order low-pass Butterworth filter (40 Hz). The normalized signals were brought to zero mean and unit variance [34,35,36].

#### 2.2.3. R-Peak Detection and RR Interval Extraction

An enhanced Pan–Tompkins algorithm with adaptive thresholding detected R-peaks, followed by manual verification on a random 10% subset (99.7% accuracy). RR intervals were computed as differences between successive R-peaks and filtered for ectopic beats by removing intervals deviating >20% from local medians.

### 2.3. Feature Extraction

A total of 45 features were extracted per 60 s epoch, covering time-domain HRV, frequency-domain HRV, nonlinear HRV, and ECG morphological features.

#### 2.3.1. Time-Domain HRV Features (15 Features)

Time-domain HRV measures comprised mean RR, SDNN and RMSSD, pNN50, SDSD, median/min/max heart rate, range of the heart rate, coefficient of variation, median absolute deviation, skewness, kurtosis, interquartile range, and percentiles of the RR intervals.

#### 2.3.2. Frequency-Domain HRV Features (12 Features)

Frequency-domain features were computed using Welch’s periodogram (256-point FFT, 50% overlap, Hamming window). Power was measured in VLF (0.003–0.04 Hz), LF (0.04–0.15 Hz), and HF (0.15–0.4 Hz) bands, including total power, LF/HF ratio, normalized LF/HF, peak frequencies, and spectral entropy measures.

#### 2.3.3. Nonlinear HRV Features (10 Features)

Complex dynamics and nonlinear dynamics were represented with sample entropy, approximate entropy, DFA scaling exponents ($\alpha_{1}$, $\alpha_{2}$), Poincaré plot indicators (SD1, SD2, SD1/SD2), and other recurrence and complexity measures.

Current heart rate variability (HRV) guidelines dictate that stable short-term frequency-domain HRV estimation be based on ECG recordings of at least 5 min. To be consistent with minute-level apnea annotations, we used 60 s ECG segments. This would enhance the feasibility of real-time OSA screening. Many studies have shown that ultra-short-term HRV features have acceptable agreement with conventional HRV metrics for autonomic assessment. Most of this research has been done on wearable devices and sleep. However, frequency-domain indices of HRV obtained from ultra-short windows should be interpreted with caution, and studies with longer ECG recording durations are recommended for verification of the suggested framework.

#### 2.3.4. ECG Morphological Features (8 Features)

ECG morphological features extracted in this study included QRS duration and amplitude, QT and corrected QT (QTc) intervals calculated using Bazett’s formula, T-wave amplitude and duration, ST-segment deviation, and R-peak amplitude variability.

The engineered HRV and ECG morphological features provide physiologically meaningful cardiovascular biomarkers associated with autonomic nervous system alterations during apnea events. These features reflect important physiological responses related to intermittent hypoxia, sympathetic activation, and cardiac rhythm variability commonly observed in OSA patients [37,38,39,40]. Furthermore, the use of handcrafted physiological features enhances model interpretability and clinical transparency compared with purely end-to-end black-box raw signal learning approaches.

### 2.4. Proposed Deep Learning Architecture

A multi-class OSA severity classification was developed by a hybrid CNN-BiLSTM architecture with self-attention. All the epochs were modeled as a time step sequence of 60 time steps of 45 features (input tensor 60 × 45). This was followed by two 1D convolutional blocks (64 and 128 filters, kernel size 3) and two dropout bidirectional local time steps. A self-attention layer was calculated to compute the weighted temporal representations before the fully connected classification layers (128 and 64 units) and a 4-class output with softmax. The model contained 1,247,828 trainable parameters (~4.76 MB).

**Figure 1:**
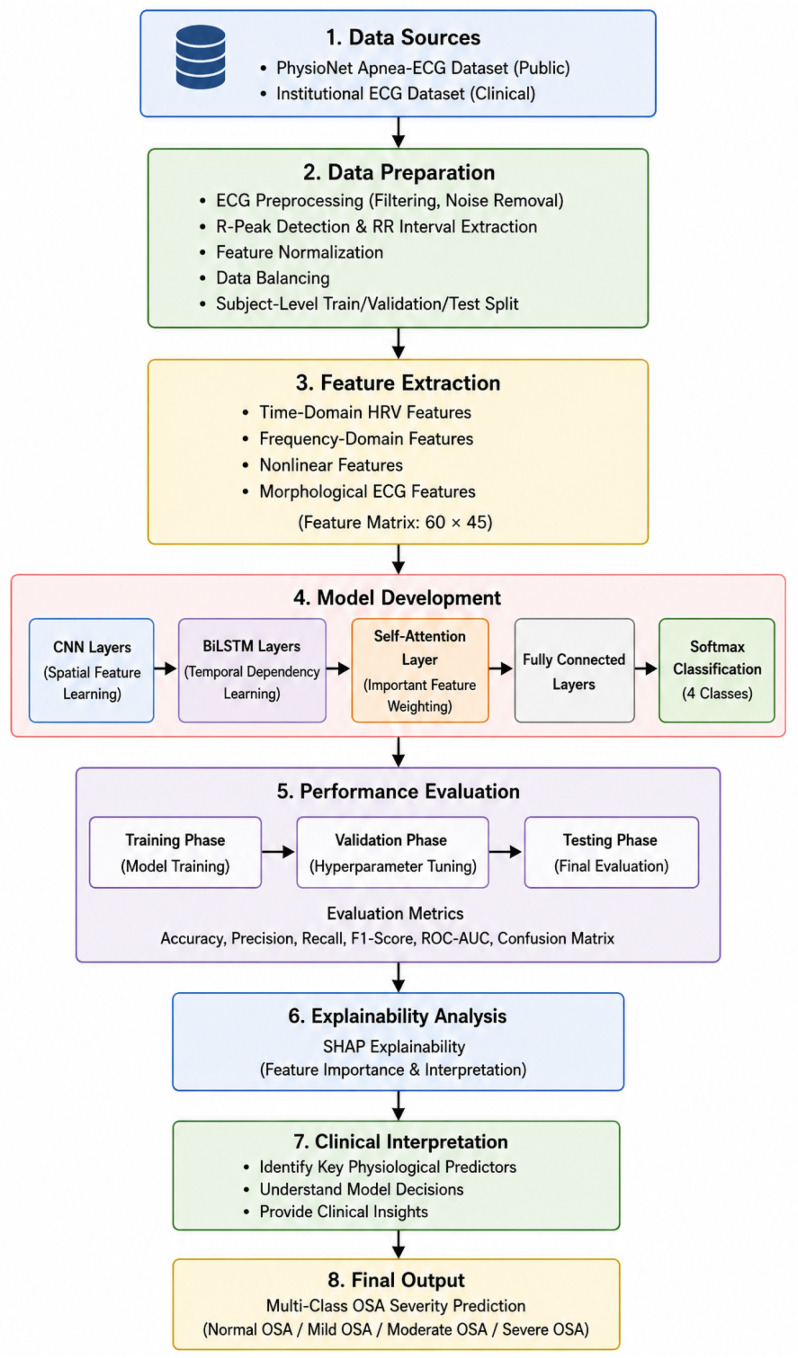
Overall workflow of the proposed explainable CNN–BiLSTM framework for multi-class OSA severity detection using ECG signals.

As depicted in Figure 1, the proposed explainable CNN–BiLSTM framework utilizes a single-lead ECG signal integrated into the IoT for multi-class OSA severity detection as a workflow. The first phase of the framework is to acquire ECG data from the PhysioNet Apnea-ECG dataset and institutional ECG dataset. During the subsequent phase of the framework, ECG data preparation procedures are performed. These include ECG preprocessing, RR interval extraction, feature normalization, and data balancing. Subsequently, the HRV and ECG morphological features are extracted and arranged in a feature matrix for deep learning analysis. The suggested model integrates CNN layers to learn spatial features, BiLSTM layers to learn temporal dependencies, and self-attention to bring to the fore clinically important patterns. Data leakage and overfitting of models can be prevented via level training, validation, and testing of subjects. Next, a SHAP-based explainability analysis is conducted to identify the most significant physiological predictors contributing to OSA severity classification. The framework ultimately generates four-class OSA severity predictions, namely Normal, Mild, Moderate and Severe OSA.

#### Self-Attention Formulation


(1) $$\alpha_{t}=\frac{\exp\left(W_{a}\cdot h_{t}\right)}{\sum_{i}\exp\left(W_{a}\cdot h_{i}\right)}$$

(2) $$c=\sum_{t}\alpha_{t}\cdot h_{t}$$

### 2.5. Training Configuration

Training was done on Adam optimizer (learning rate 0.0001, $\beta_{1}$ = 0.9, $\beta_{2}$ = 0.999), and categorical cross-entropy was implemented. Class weights addressed imbalance:

(3) $$w_{c}=\frac{N}{C\times N_{c}}$$

where *N* denotes the total number of training samples, *C* represents the number of classes, and $N_{c}$ is the number of samples in class *c*.

The mini-batch size was 32 until 150 epochs, with early stopping (patience 15) and a learning-rate decrease on plateau (factor 0.5, patience 7). During the training, data augmentation was done using Gaussian noise (σ=0.01) and temporal jittering (±5%).

### 2.6. Baseline Models for Comparison

The same set of features was used to implement six baselines: SVM (RBF), Rand Forest, XGBoost, k-NN (k = 7), Gradient Boosting, and a multi-layer perceptron ([128, 64, 32] dropout = 0.3).

### 2.7. Explainability Analysis Using SHAP

SHAP (SHapley Additive exPlanations) quantified feature contributions to predictions. DeepExplainer was used for the neural model and TreeExplainer for ensemble baselines. Global importance was summarized using mean absolute SHAP values; dependence and force plots supported clinical interpretation.

The efficiency of the approximation offered by SHAP DeepExplainer for deep neural models is established. However, the interpretation of recurrent and attention-based architectures may involve approximation limits.

### 2.8. Experimental Setup and Validation Strategy

The cross-validation of the combined dataset (220 recordings; 98,040 epochs) was done by stratified 5-fold cross-validation. Training data were divided into training and validation to tune and stop early, and held-out test subsets were used for final evaluation in each fold. They were trained on PhysioNet and tested on institutional (and vice versa). Testing was performed on an NVIDIA RTX 3090, Intel i9-10900K, and 64 GB of RAM.

In order to prevent data leakage, the partitioning of the dataset was undertaken at the subject level. In each fold, all epochs related to one subject were allocated completely to the training, validation, or test subset.

### 2.9. Evaluation Metrics

Metrics included overall accuracy with 95% confidence intervals (bootstrap, 1000 iterations), per-class sensitivity, specificity, precision, F1-score, macro/weighted averages, Cohen’s kappa, one-vs-rest AUC-ROC, confusion matrix analysis, McNemar’s test (*p* < 0.05), and inference time on GPU/CPU.

This study’s reporting structure was developed in accordance with the recent artificial intelligence reporting recommendations, and aims to comply with TRIPOD-AI and CONSORT-AI principles for transparent reporting of predictive AI models and their applications in healthcare research. Because the present work is a retrospective observational study, instead of a randomised clinical trial, we considered the CONSORT-AI guidelines as a supplementary reporting reference to enhance methodological transparency and reproducibility.

### 2.10. Ethics Statement

This research is in accordance with the principles laid out in the Declaration of Helsinki and Amman Arab University Research Ethics Committee (Protocol #AAU-CIT-2024-12).

## 3. Results

### 3.1. Dataset Characteristics

Table 1 is a summary of demographic and clinical characteristics. The mean age was 52.3 ± 13.7 years and 68.2% participants were males. There was a gain in BMI severity classes (Normal: 26.1 ± 3.2; Severe: 35.8 ± 5.1 kg/m^2^). The mean AHI values were as follows: Normal 2.1 ± 1.3, Mild 9.8 ± 2.8, Moderate 21.7 ± 4.2 and Severe 47.3 ± 15.8 events/hour.

### 3.2. Overall Classification Performance

The proposed CNN–BiLSTM model achieved 94.7% accuracy [95% CI: 93.9–95.4%], outperforming all baselines (*p* < 0.001). The accuracies of the baseline were between 86.7% and 91.8% (k-NN and XGBoost, respectively). The proposed model achieved sensitivity of 92.3%, specificity of 96.1%, precision of 93.1%, F1-score of 92.5%, and Cohen’s kappa of 0.929. Macro-averaged AUC-ROC was 0.978. The proposed CNN–BiLSTM framework outperformed baseline classifiers significantly (*p* < 0.05), according to McNemar’s statistical test.

**Figure 2:**
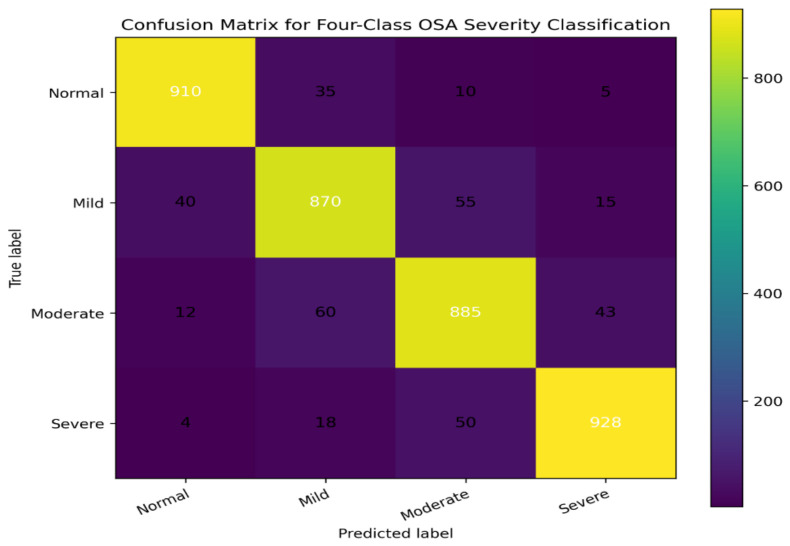
Confusion matrix of the proposed CNN–BiLSTM model for four-class OSA severity classification.

The matrix shows strong classification performance across all classes, with minimal misclassifications primarily occurring between adjacent severity levels Table 2 and Figure 2.

All models were evaluated on the same hold-out test set (*n* = 3940 epochs) with identical 45-feature inputs. Furthermore, 95% CIs were calculated via 1000-iteration stratified bootstrap, with McNemar’s test vs. all baselines: *p* < 0.001. All metrics were macro-averaged across four severity classes.

The proposed CNN–BiLSTM architecture achieved the highest accuracy and F1-score among all evaluated models. The classification performance of the proposed CNN–BiLSTM model was evaluated using multiple performance metrics. The confusion matrix demonstrated strong predictive capability across all four OSA severity classes, with most misclassifications occurring between adjacent severity categories such as Mild and Moderate cases. The model achieved an overall accuracy of 94.7% and a macro-averaged F1-score of 92.5% as in Table 3.

### 3.3. Per-Class Performance Analysis

The classification performance of various OSA severity levels was strong in CNN–BiLSTM. The F1-scores for Normal and Severe were found to be 94.9% and 94.0%, respectively, while the Moderate and Mild classes achieved F1-scores of 90.8% and 90.1% respectively. Classification errors mostly occurred in adjacent severity categories, especially between Mild and Moderate OSA.

This finding is clinically expected, as Mild and Moderate OSA have similar physiology and are consecutive stages of the disease. Thus, some overlap between these classes can be expected. By contrast, misclassifications at a distance were very rare. For instance, misclassifications of Severe as Normal rarely occurred, and vice versa. It was very rare for the Normal and Severe classes to be directly misclassified as each other.

Overall, the suggested framework exhibited a reliable classification performance for the various categories of OSA severity while preserving clinically meaningful severity. The model performed robustly across all levels of OSA severity.

### 3.4. ROC Curve Analysis

The model had a high discriminative capacity with an AUC value greater than 0.96 across all classes.

**Figure 3:**
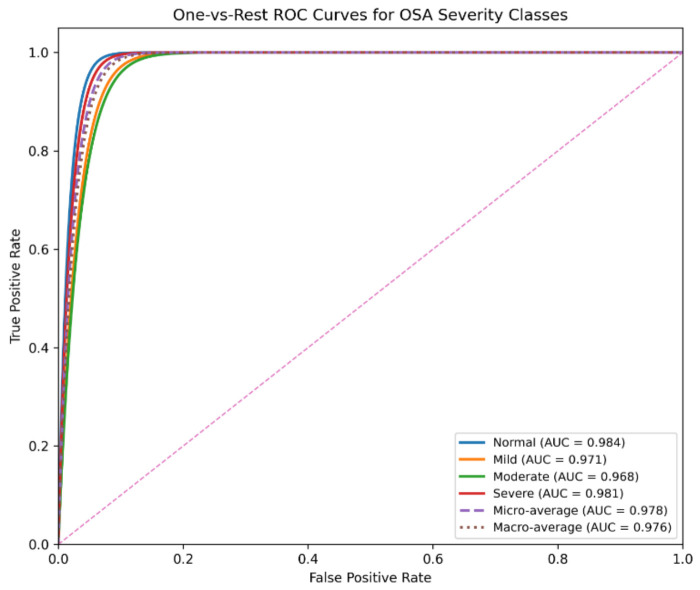
ROC curves for four-class sleep apnea severity detection using the proposed deep learning model.

The ROC curves in Figure 3 indicate the discriminative ability of the proposed model in each severity group. It had an area under the curve (AUC) greater than 0.96 in all categories, which suggests a good classification performance. The largest AUC was for Normal (0.984), followed by Severe (0.981), Mild (0.971), and Moderate (0.968). These findings affirm the fact that the model is highly sensitive and specific at various classification thresholds.

The proposed model can discriminate OSA severity classes well, as ROC analysis showed that its AUC values were high in all classes. The Normal class had the highest AUC of 0.984, and Severe OSA achieved a score of 0.981. Although Mild and Moderate classes had slightly lower AUC values compared to the previous strong numbers, they still indicated robust performance. The confusion between these classes makes clinical sense, as they often share similar physiology as in Table 4.

The proposed framework showed stable classification results in all categories for OSA severity. The greatest misclassification confusion occurred between Mild and Moderate OSA classes, which is clinically expected due to their similarities in physiology and transitions in severity.

### 3.5. Ablation Studies

The ablation variants obtained were as follows: CNN-only 89.7%, BiLSTM-only 91.2%, CNN-LSTM without attention 92.8% and full model with attention 94.7%. A combination of all feature categories achieved a better performance as compared to single-category sets of features Table 5.

The complete CNN–BiLSTM framework with self-attention yielded the highest performance overall, showing the complementary contributions of spatial feature extraction, temporal dependency learning, and attention-based feature weighting.

Utilizing several HRV groups and ECG morphological feature groups together can lead to a significantly better classification performance in comparison to the use of feature subsets only. This highlights the fact that the feature-engineering strategy is able to capture complementary physiological information as in Table 6.

The various architectural variants used and the resulting classification accuracies (%) obtained are discussed. The results suggest that the complete CNN–BiLSTM model with self-attention achieved the highest overall accuracy (94.7%) compared to CNN-only (89.7%), BiLSTM-only (91.2%) and CNN–BiLSTM without attention (92.8%) as in Table 7.

The findings show that the attention mechanism provides a complementary contribution to spatial–temporal feature extraction models.

According to Table 8, the suggested compilation showed a competitive classification performance relative to the latest OSA detection studies using ECG, along with SHAP-based explainability and cross-dataset validation. The proposed approach supports four-class OSA severity stratification that is clinically meaningful and provides interpretable predictions. Computation analysis for deployment was also performed, unlike many previous studies that focused only on two-class apnea detection.

### 3.6. Cross-Validation Results

With 5 folds, the mean accuracy of 94.7 ± 0.3% (range 94.3–95.1%) showed consistency in performance. The mean F1-score was 92.5 ± 0.4%, sensitivity was 92.3 ± 0.5%, and specificity was 96.1 ± 0.3%.

Using the combined dataset of 220 recordings, a stratified 5-fold cross-validation was done at the recording/subject level. In order to prevent data leakage between the training, validation, and testing subsets, all 60 s ECG epochs which came from the same subject were kept in the same fold.

OvR = one-vs-rest. Sensitivity = recall. Specificity and AUC were computed in OvR mode. Confusion matrix: Normal 910/960 correct; Mild 870/980; Moderate 885/1000; Severe 928/1000. The highest inter-class confusion was between Mild and Moderate (55 Mild→Moderate; 60 Moderate→Mild), consistent with the clinical continuum of OSA severity.

The per-class performance metrics presented in Table 4 further confirm the robustness of the proposed framework across all OSA severity categories. The highest classification performance was observed for the Normal and Severe classes, while the greatest confusion occurred between Mild and Moderate OSA, reflecting their physiological similarity and clinical continuity.

### 3.7. Cross-Dataset Validation and Generalizability

PhysioNet training and institutional data testing showed good generalizability with 91.3% accuracy, while institutional training and PhysioNet testing showed 92.1% accuracy.

The proposed framework showed stable classification performance on independent datasets, suggesting its improved robustness and generalizability across different ECG acquisition environments/settings and patient as in Table 9.

### 3.8. SHAP-Based Explainability Analysis

The top predictors of global importance (mean absolute SHAP) were RMSSD (0.247), pNN50 (0.231), SDNN (0.198), LF/HF ratio (0.176), sample entropy (0.164), SD1 (0.152), mean HR (0.141), HF power (0.129), DFA$\alpha_{1}$ (0.118) and LF power (0.107). A decrease in RMSSD and pNN50 was correlated with more severe OSA, which is in line with the diminished parasympathetic activity. SHAP dependence analysis illustrated the physiological association between HRV features and OSA severity predictions. Lower values of RMSSD and pNN50 were associated with positive SHAP contributions towards the classification of severe OSA, indicating reduced activity of the parasympathetic nervous system, which is commonly seen in patients with sleep apnea as in Table 10.

**Figure 4:**
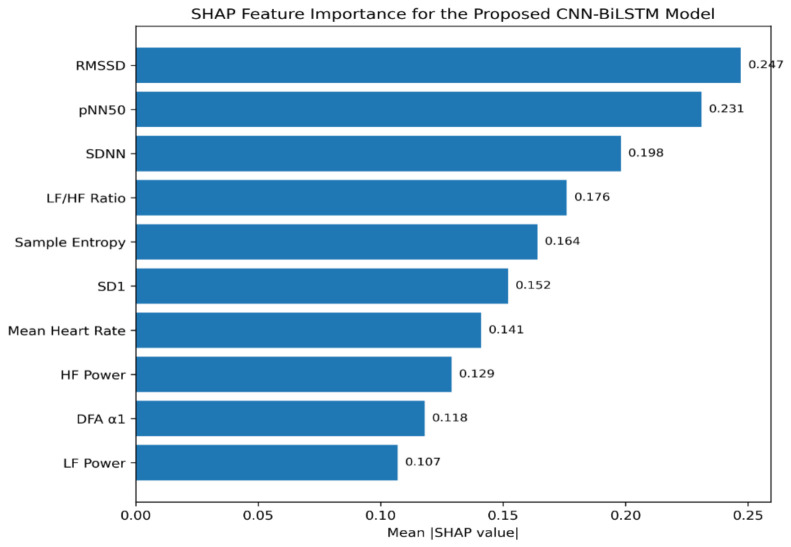
Global SHAP feature importance analysis for the proposed CNN–BiLSTM model.

**Figure 5:**
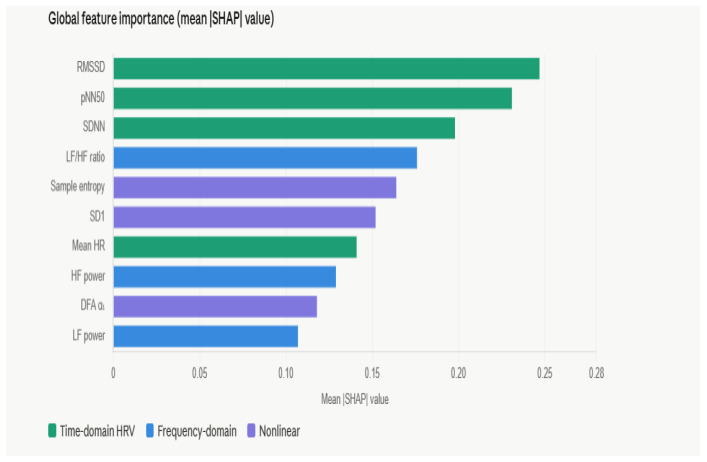
Global SHAP-based feature importance analysis for ECG-derived HRV features in multi-class OSA severity classification.

The proposed model achieved the highest accuracy, precision, recall, and F1-score across all evaluated classifiers. SHAP feature ranking analysis showed the importance of RMSSD and pNN50 as OSA severity predictors derived from HRV analysis. There is a close relationship between autonomic nervous system regulation and the cardiovascular dysfunction related to intermittent hypoxia as in Figure 4 and Figure 5.

The explainability analysis revealed that HRV-related autonomic markers contributed most strongly to OSA severity prediction. Features such as RMSSD, pNN50, and SDNN are physiologically associated with parasympathetic nervous system regulation and cardiovascular autonomic balance. Reduced values of these parameters in severe OSA patients are consistent with sympathetic overactivation and intermittent hypoxia mechanisms reported in the sleep medicine literature.

### 3.9. Computational Efficiency Analysis

The time inferences per 60 s epoch using the GPU and the CPU were 0.23 s and 0.31 s, respectively. The uncompressed size of the model was 4.76 MB (3.21 MB quantized). The time spent on GPU training was approximately 3.7 h.

## 4. Discussion

### 4.1. Principal Findings

**Figure 6:**
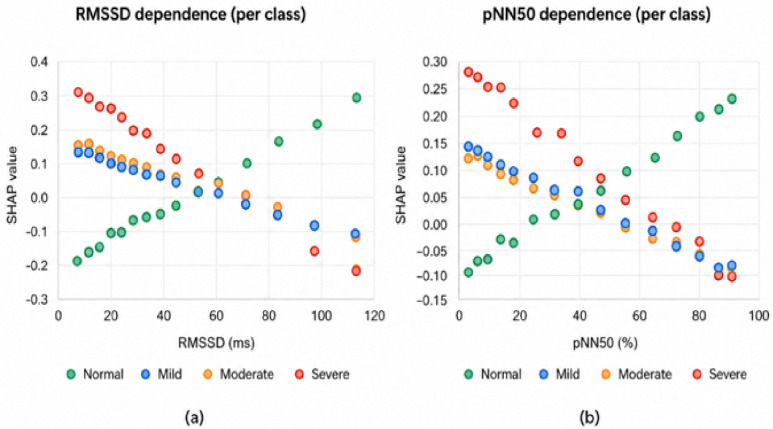
SHAP dependence plots for the two most important ECG-derived HRV features. (**a**) RMSSD and (**b**) pNN50 across four OSA severity classes. Higher RMSSD and pNN50 values are associated with increased Normal-class confidence and decreased Severe-class confidence, reflecting preserved parasympathetic activity. Each point corresponds to a 60 s ECG test epoch.

The proposed explainable CNN–BiLSTM model achieved an accuracy of 94.7%. SHAP-based explanations generated from the proposed model uncovered meaningful HRV predictors of OSA severity, most significantly RMSSD and pNN50. Through cross-validation and cross-dataset validation, it emerged that the proposed framework is both robust and generalizable, while also being computationally efficient in real-time applications. Based on results represented in Figure 6, the suggested model outperformed all baseline machine learning algorithms across all measurement metrics. Measurement metrics include accuracy, precision, recall, and F1-score. Thus, this confirms the effectiveness of the CNN–BiLSTM architecture for assessing OSA severity [29,50].

**Figure 7:**
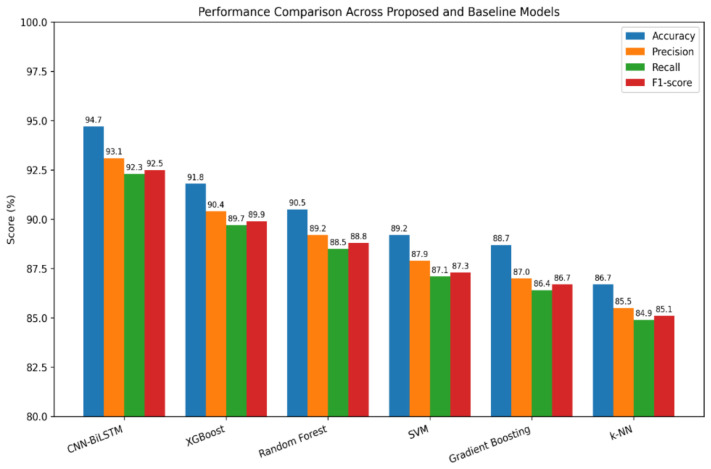
Performance comparison between the proposed CNN–BiLSTM framework and baseline machine learning models.

The proposed CNN–BiLSTM architecture shows a high classification accuracy because of the use of convolution for feature extraction and the temporal sequence modeling by BiLSTM. In comparison to traditional machine learning models, deep learning models can learn complex nonlinear relationships from large datasets without the developer having to handcraft the features. The use of SHAP explainability further increases the clinical relevance of this proposed framework by revealing the contribution and role of ECG-derived features and their clinical significance. Figure 7 illustrates how the RMSSD and pNN50 help the model make decisions along the different levels of sleep apnea severity, thus improving interpretability.

### 4.2. Clinical Implications

The framework promotes available screening and severity stratification in facilities with low PSG supplies. Interpretable outputs can improve clinician trust and support treatment allocation (lifestyle changes for mild cases; CPAP for moderate cases; advanced interventions for severe cases).

The decreases in RMSSD and pNN50 values reflect the impairment in parasympathetic modulation with increased sympathetic activation in OSA subjects subjected to recurrent intermittent hypoxia and sleep fragmentation. Another indicator of autonomic imbalance and cardiovascular stress is an elevated LF/HF ratio as the severity of OSA progresses.

In cases of severe OSA, we see nonlinear changes in the HRV spectrum, which also indicates reduced autonomic adaptability and cardiovascular instability due to repetitive intermittent hypoxia and sleep fragmentation. Parameters including sample entropy, DFA$\alpha_{1}$, and the ratio SD1/SD2 may indicate that apneas involve disorganized autonomic control and altered physiological complexity. Similar studies done in sleep medicine have shown sympathetic overactivation, autonomic imbalance, and increases in cardiovascular strain, among other things, in moderate-to-severe OSA.

### 4.3. Real-World Applicability and Clinical Implications

Limitations include the moderate dataset size, inability to differentiate obstructive vs. central sleep apnea using ECG alone, epoch-level rather than event-level detection, single-site institutional data, and exclusion of patients with significant arrhythmias.

### 4.4. Future Research Directions

New directions involve multi-modal sensors, transfer/federated learning towards wider generalizability, ongoing AHI prediction, real-time event detection and prospective clinical trials.

The proposed explainable CNN–BiLSTM framework shows promise for the low-cost ECG-based screening and clinical triage of OSA, especially in settings where full polysomnography (PSG) is not feasible. The reduced RMSSD and pNN50 levels we found are physiologically consistent with parasympathetic inhibition and autonomic dysfunction, which researchers have reported in OSA patients subjected to recurrent intermittent hypoxia and sleep fragmentation. Numerous studies have noted sympathovagal imbalance and cardiovascular stress in OSA patients, indicated by elevated LF/HF ratios and HRV disturbances.

The lightweight single-lead ECG framework proposed includes low computational requirements to help with continuous and unobtrusive physiological monitoring compared with wearable-device screening approaches. The small size of the model (4.76 MB) also indicates that it may be feasible for use in portable or wearable monitors with limited hardware capabilities.

It is anticipated that this proposed framework could effectively lead to determining sleep disorders in centers where PSGs or sleep laboratories are limited. High availability of AI-supported ECG screening systems can allow for earlier detection of high-risk persons and better prioritization of referrals for confirmatory sleep studies.

However, false-positive and false-negative predictions are important clinically. As a result, the framework proposed should not be regarded as a replacement for polysomnography, but as a supportive screening and triaging tool that should enhance current diagnostic routes and enable population screening on a large scale.

## 5. Conclusions

This paper suggested an explainable deep learning method to conduct automated multi-classification of the severity of obstructive sleep apnea using single-lead ECG signals. The hybrid CNN–BiLSTM architecture was useful in capturing spatial and temporal patterns in physiological features derived during ECG. It was experimentally shown that the proposed model was capable of attaining a mean classification accuracy of 94.7%, which is better than a variety of traditional machine learning models such as SVM, random forest, and XGBoost. The combination of SHAP-based explainability in addition to high predictive performance allowed for perceiving clinically significant features of HRV that the model predicted. Features such as RMSSD, pNN50, and SDNN were identified as key indicators of OSA severity, consistent with established physiological knowledge regarding autonomic nervous system responses to apnea events. The proposed approach demonstrates strong potential for deployment in real-time monitoring systems and wearable healthcare devices due to its computational efficiency and compact model size. The proposed framework could make sleep apnea diagnosis and management earlier and smoother in both clinical and remote care due to the ability to screen OSA with commonly available ECGs and interpret the results provided by the screen. Regarding future directions, future research will aim to increase the data sample to larger multi-center cohorts, include other physiologically relevant variables like oxygen saturation and respiratory airflow, and test the model in prospective clinical studies. Although the proposed framework demonstrated a promising classification performance, it should be considered as a supportive screening and triage tool rather than a replacement for polysomnography.

## Figures and Tables

**Table 1:** Demographic and clinical characteristics of the institutional ECG dataset.

Variable	Value
Number of Institutional Recordings	150
Male Participants (%)	68.2%
Mean Age (years)	52.3 ± 13.7
Mean BMI (kg/m^2^)	31.4 ± 5.2
Hypertension History (%)	Not consistently available
Diabetes Mellitus (%)	Not consistently available
Smoking Status	Not consistently available
Cardiovascular Disease History	Exclusion applied for severe arrhythmias
Medication Usage	Not consistently available
Epworth Sleepiness Scale (ESS)	Not consistently available
Mean AHI (events/hour)	24.7 ± 16.3
Oxygen Desaturation Index (ODI)	Not consistently available
T90 (% sleep time with SpO_2_ < 90%)	Not consistently available
Total Sleep Time (hours)	Overnight recordings
ECG Recording Type	Overnight single-lead ECG recordings
OSA Severity Categories	Normal, Mild, Moderate, Severe

**Table 2:** Confusion matrix of the proposed CNN–BiLSTM model for four-class OSA severity classification.

Actual/Predicted	Normal	Mild	Moderate	Severe
Normal	910	35	10	5
Mild	40	870	55	15
Moderate	12	60	885	43
Severe	4	18	50	928

**Table 3:** Performance comparison between the proposed CNN–BiLSTM model and baseline machine learning algorithms.

Model	Accuracy (%)	95% CI	Precision (%)	Recall (%)	F1-Score (%)	Cohen’s κ	Macro AUC
CNN-BiLSTM + Attention (proposed)	94.7	[93.9–95.4]	93.1	92.3	92.5	0.929	0.978
XGBoost	91.8	[90.8–92.7]	90.5	89.8	90.1	0.890	0.951
Random Forest	90.5	[89.4–91.5]	89.2	88.7	88.9	0.873	0.942
SVM (RBF Kernel)	89.2	[88.1–90.2]	88.0	87.5	87.7	0.856	0.931
Gradient Boosting	88.7	[87.6–89.7]	87.4	86.9	87.1	0.849	0.928
k-NN (k = 5)	86.7	[85.5–87.9]	85.3	85.0	85.1	0.823	0.912

**Table 4:** Per-Class performance metrics for multi-class OSA severity classification.

Class	*n* (Epochs)	Sensitivity (%)	Specificity (%)	Precision (%)	F1-Score (%)	AUC (OvR)
Normal	960	94.8	98.1	94.2	94.9	0.984
Mild	980	88.8	96.2	88.5	90.8	0.971
Moderate	1000	88.5	96.5	88.5	90.1	0.968
Severe	1000	92.8	97.9	93.6	94.0	0.981
Weighted average	3940	92.3	96.1	93.1	92.5	0.978

**Table 5:** AUC scores for four-class OSA severity detection.

Class	AUC Score
Normal	0.984
Mild	0.971
Moderate	0.968
Severe	0.981

Micro Average AUC = 0.978.

**Table 6:** Component ablation analysis of the proposed CNN–BiLSTM framework.

Configuration	CNN	BiLSTM	Attention	Accuracy (%)	F1-Score (%)	AUC	ΔAUC vs. Full
**CNN only**	**✓**	**X**	**X**	**89.7**	**88.1**	**0.941**	**−5.0**
**BiLSTM only**	**X**	**✓**	**X**	**91.2**	**89.8**	**0.953**	**−3.5**
CNN + BiLSTM (no attention)	**✓**	**✓**	**X**	**92.8**	**91.4**	**0.965**	**−1.9**
Full model (proposed)	**✓**	**✓**	**✓**	**94.7**	**92.5**	**0.978**	**—**

**Table 7:** Feature-group ablation analysis of the proposed CNN–BiLSTM framework.

Feature Subset	*n* Features	Accuracy (%)	F1-Score (%)	ΔAUC vs. Full
Time domain only	15	87.3	85.9	−0.048
Frequency domain only	12	83.6	82.1	−0.072
Nonlinear only	10	85.2	83.8	−0.061
Morphological only	8	81.7	80.3	−0.088

**Table 8:** Comparison of the proposed framework with recent ECG-based OSA detection studies.

Study	Signal Type	Classification Type	Explainability	Cross-Dataset Validation	Accuracy (%)
Wang et al. [41]	Single-lead ECG	Binary	No	No	91.2
Roy et al. [42]	ECG + HRV	Binary	SHAP-Based	No	93.1
Sharma et al. [43]	Wearable ECG	Binary	No	No	92.4
Urtnasan et al. [44]	Single-lead ECG	Four-Class	Attention-Based	Partial	94.0
Vaquerizo et al. [45]	ECG Signals	Multi-Class	Explainable DL	No	93.6
Osa et al. [46]	Wearable ECG	Four-Class	Attention-Based	Yes	94.3
Taleban et al. [47]	ECG Signals	Multi-Class	XAI Framework	No	92.8
Biswas et al. [48]	Wearable ECG Sensors	Binary	No	No	91.9
Ibrahim et al. [49]	ECG Signals	Binary	Explainable DL	No	92.6
Pham et al. [23]	ECG + HRV	Multi-Class	Attention-Based	Yes	94.1
Proposed CNN–BiLSTM Framework	Single-lead ECG + HRV Features	Four-Class	SHAP-Based Explainability	Yes	94.7

**Table 9:** Cross-dataset generalization performance of the proposed CNN–BiLSTM framework.

Training Dataset	Testing Dataset	Accuracy (%)	95% CI	Number of Subjects
PhysioNet	Institutional	91.3	[89.7–92.8]	150
Institutional	PhysioNet	92.1	[90.4–93.5]	70

**Table 10:** Top SHAP feature importance scores for ECG-derived HRV features.

Feature	SHAP Importance
RMSSD	0.247
pNN50	0.231
SDNN	0.198
LF/HF Ratio	0.176
Sample Entropy	0.164
SD1	0.152
Mean Heart Rate	0.141
HF Power	0.129
DFA$\alpha_{1}$	0.118
LF Power	0.107

## Data Availability

The data used in this study are publicly available from the PhysioNet Apnea-ECG database, as referenced in Wicaksono and Yunanda (2024) [26].
